# Losing genes, gaining edits: how relaxed selection and inverted repeat expansion shape RNA editing in Schizaeaceae plastomes

**DOI:** 10.1111/tpj.70919

**Published:** 2026-06-12

**Authors:** Blake D. Fauskee, Li‐Yaung Kuo, Farley Kwok van der Giezen, Kathleen M. Pryer

**Affiliations:** ^1^ Department of Biology Duke University Durham 27708 North Carolina USA; ^2^ Institute of Molecular and Cellular Biology National Tsing Hua University Hsinchu Taiwan; ^3^ ARC Centre of Excellence in Plant Energy Biology, School of Molecular Sciences The University of Western Australia Perth Western Australia 6009 Australia

**Keywords:** chloroplast, genomics, relaxed selection, RNA editing, Schizaeaceae

## Abstract

RNA editing is a post‐transcriptional pyrimidine exchange process that alters plastid and mitochondrial transcripts in nearly all land plants. Although confined to organelles, it is directed by nuclear‐encoded PLS‐type pentatricopeptide repeat (PPR) proteins, each typically recognizing a specific RNA target. While many editing sites are functionally neutral, edits at cryptic start and internal stop codons have been implicated in modulating organellar gene expression. Ferns—and some lycophytes—are unique among vascular plants in exhibiting both C‐to‐U and U‐to‐C editing, making them valuable for studying the evolution of both forms. Here, we examine chloroplast RNA editing in four Schizaeales species (*Schizaea dichotoma*, *Actinostachys digitata*, *Anemia phyllitidis*, *Lygodium microphyllum*). *Schizaea* and *Actinostachys* possess non‐photosynthetic gametophytes, providing a natural contrast with fully photosynthetic relatives. Despite extensive plastome reduction, including loss of the *ndh* suite and, in *Actinostachys*, all *chl* genes, *Schizaea* and *Actinostachys* exhibit dramatically elevated numbers of C‐to‐U edits. Genes evolving under relaxed selection accumulate more editing sites, and editing abundance per gene correlates with the magnitude of relaxed constraint, suggesting relaxed selection promotes edit proliferation. *Schizaea dichotoma* and *A. digitata* also show expansion of the chloroplast inverted repeat (IR), and genes translocated into the IR exhibit reduced substitution rates and higher editing densities, indicating that IR expansion slows the loss of edits. Finally, annotation of PPR proteins revealed few full‐length editing factors, consistent with catalytic domains assembling *in trans* and highlighting the modular nature of the fern editosome.

## Significance Statement

To understand how chloroplast RNA editing evolves in the context of plastome degradation and reduced photosynthetic capacity, we examined ferns in the order Schizaeales, some of which have non‐photosynthetic gametophytes and highly restructured plastomes. By contrasting partially non‐photosynthetic and fully photosynthetic species, we show that relaxed purifying selection promotes the rapid accumulation of C‐to‐U—but not U‐to‐C—edits, while inverted repeat expansions further preserve existing edits.

## INTRODUCTION

Nearly all land plants undergo RNA editing, a post‐transcriptional process that introduces targeted nucleotide modifications to RNA transcripts derived from organellar genomes. This editing process yields mRNA sequences that no longer match their DNA templates (reviewed in Knoop, 2023). Most RNA editing events in organellar genomes act to restore evolutionarily conserved amino acid sequences to faithfully yield functional proteins (reviewed in Small et al., 2020). Two forms of RNA editing occur in land plant organelles: C‐to‐U RNA editing, where cytidines are deaminated to yield uridines, and U‐to‐C editing, where uridines are converted to cytidines via a suspected transamination reaction (reviewed in Chateigner‐Boutin & Small, 2011; Ichinose & Sugita, 2017; Hayes et al., 2024). C‐to‐U editing restores protein functionality in the organellar genomes of all land plants, except for the liverwort *Marchantia polymorpha* (Rüdinger et al., 2008; Shen et al., 2024). U‐to‐C editing presents a different evolutionary picture where it is absent in seed plants (Tillich et al., 2006), but retained in hornworts (Kugita et al., 2003), some lycophyte lineages (Grewe et al., 2011; Kwok van der Giezen et al., 2024), and ferns (Fauskee et al., 2025; Guo et al., 2015; Wolf et al., 2004).

Although RNA editing in plants occurs primarily in organellar genomes, it is carried out by nuclear‐encoded pentatricopeptide repeat (PPR) proteins (Chateigner‐Boutin et al., 2008; Lurin et al., 2004). PPR proteins are a large family of RNA‐binding proteins that play diverse roles in organellar RNA metabolism, including editing, transcript stabilization (Prikryl et al., 2011), splicing (Chateigner‐Boutin & Small, 2011; de Longevialle et al., 2007), and endonucleolytic cleavage (Huynh et al., 2023). These proteins are composed of tandem arrays of helix‐turn‐helix units, known as PPR motifs, that form a super‐helical structure with the central RNA‐binding groove. Individual PPR motifs typically recognize one RNA nucleotide at a time, with binding specificity conferred by hydrogen bonds formed between the fifth and C‐terminal amino acid residues in each PPR motif and the corresponding RNA base (Barkan et al., 2012). PPR proteins are present throughout eukaryotes, but the gene family has undergone dramatic expansion in land plants, where these proteins have been co‐opted for highly specialized roles in organellar gene expression (Gutmann et al., 2020). Land plant PPR proteins are broadly divided into two classes based on their motif architecture and function. P‐class PPR proteins consist of tandem repeats of a 35 amino acid motif and primarily function in RNA stabilization, splicing, and cleavage. In contrast, PLS‐class PPR proteins function as RNA editing enzymes and contain repeating triplet units of P1 (35 amino acids), L1 (35 amino acids), and S1 (31 amino acids) motifs, often accompanied by additional P2 (35 amino acids), L2 (36 amino acids), and S2 (32 amino acids) variants. These proteins are typically capped by C‐terminal catalytic domains—either E1‐E2‐DYW for C‐to‐U editing, or E1‐E2‐DYW:KP for U‐to‐C editing—that carry out the nucleotide base conversions necessary for producing functional transcripts.

Although some RNA editing sites—notably C‐to‐U edits that restore start codons and U‐to‐C edits that correct internal stop codons—are evolutionarily conserved for their adaptive roles in regulating plastid gene expression (Fauskee et al., 2025), most editing sites likely evolve through a neutral evolutionary ratchet mechanism known as constructive neutral evolution (CNE). This is where otherwise deleterious mutations in organellar DNA are tolerated and reach fixation because they are post‐transcriptionally corrected through RNA editing (Covello & Gray, 1993; Lukeš et al., 2011). Under the CNE model, new RNA editing sites proliferate when organellar genome mutations are compensated by preexisting nuclear‐encoded PLS‐class PPR editing proteins, which post‐transcriptionally restore protein function through editing. Despite the potential for RNA editing sites to accumulate neutrally, several comparative studies have shown progressive loss of editing sites in the chloroplast genomes of angiosperms (Ishibashi et al., 2019; Mower, 2008) and several fern lineages (Fauskee et al., 2025). This trend likely reflects mutational bias in AT‐rich chloroplast genomes, which favors loss of C‐to‐U editing sites, the only editing type in angiosperms and the predominant type in ferns. C‐to‐T transitions occur at a particularly high rate in chloroplast genomes (Huang et al., 2005). When these mutations arise at C‐to‐U editing sites, they restore the ancestral codon sequence, thereby eliminating the need for editing at that position. Thus, while a small subset of RNA editing sites is conserved by selection, the overall evolutionary trend of RNA editing in most plant lineages is one of neutral gain outpaced by progressive loss.

Despite recent advances in our understanding of plastid RNA editing (Fauskee et al., 2025; Gerke et al., 2020; Guo et al., 2015; Ishibashi et al., 2019; Small et al., 2020; Wolf et al., 2004), little is known about how this process operates in plants exhibiting partial or complete heterotrophy during their life cycle. Likewise most research on plastome evolution has centered on green plants with highly conserved plastome architecture (Mower & Vickrey, 2018). With few exceptions, land plant plastomes are remarkably stable in size, structure, and gene content: most are circular‐mapping molecules (Rochaix, 1978) ranging from 120 to 160 kb in length and exhibit a quadripartite structure consisting of a large and small single‐copy region (LSC and SSC, respectively) separated by two inverted repeats (IR_A_ and IR_B_) (Mower & Vickrey, 2018; Ruhlman & Jansen, 2014). Plastomes of fully autotrophic plants typically encode approximately 80 protein‐coding genes (Wicke et al., 2011), four rRNAs (consistently located in the IR; Zhu et al., 2016), and around 30 tRNAs (Jansen & Ruhlman, 2012). In contrast, plastomes of non‐photosynthetic plants—where both the sporophyte and gametophyte lack photosynthetic ability—and partially non‐photosynthetic plants—where only one life stage retains photosynthesis—frequently exhibit significant departures from this conserved genomic organization. Research on independently derived parasitic and mycoheterotrophic lineages has revealed extensive and rapid gene loss (particularly *ndh* genes), genome size reduction, and structural rearrangements, primarily driven by relaxed purifying selection on genes associated with photosynthesis (Barrett et al., 2014; Bellot & Renner, 2016; Wicke et al., 2016). These pronounced differences raise an important and largely unexplored question: how does plastid RNA editing evolve under conditions of plastome deterioration and diminished selective pressure on photosynthetic function?

To the best of our knowledge, there is only one study to date that has investigated the plastid editome of a non‐photosynthetic plant. Funk et al. (2007) identified plastid RNA editing sites in two parasitic species of *Cuscuta*—*C. reflexa* and *C. gronovii*—revealing reduced editing levels in both relative to their close photosynthetic relatives. While *Cuscuta* represents an important case of plastome degradation and functional gene loss associated with a parasitic lifestyle, the utility of angiosperms for broader evolutionary studies of plastid RNA editing is limited in that they exclusively possess C‐to‐U editing and retain relatively few editing sites—typically 30–50 per plastome (reviewed in Small et al., 2020). This limits investigation of evolutionary patterns in the diversity, directionality, and abundance of plastid RNA editing.

Ferns and hornworts, by contrast, are the two most extensively studied plant lineages exhibiting abundant plastid RNA editing, with both C‐to‐U and U‐to‐C editing types present (Fauskee et al., 2025; Guo et al., 2015; Kugita et al., 2003; Villarreal et al., 2018). Ferns are unique among land plants in that they have free‐living sporophyte and gametophyte generations, each with distinct physiological roles (reviewed in Krieg & Chambers, 2022). In almost all fern lineages, both life stages are photosynthetically active. The Schizaeaceae (order Schizaeales) are exceptional; several groups produce achlorophyllous gametophytes, while members of the other families in the order—Anemiaceae and Lygodiaceae—retain green gametophytes (Bierhorst, 1975). The plastomes of Schizaeaceae exhibit strikingly similar genomic reductions to those seen in heterotrophic angiosperms: all members have lost the entire suite of plastid‐encoded NAD(P)H dehydrogenase complex (*ndh*) genes, while *Actinostachys* has additionally lost the light‐independent protochlorophyllide reductase (*chl*) genes (Ke et al., 2022; Labiak & Karol, 2017). This well‐characterized variation in gametophyte plastid function and plastome structure in Schizaeales provides a powerful comparative framework for examining how both C‐to‐U and U‐to‐C RNA editing evolves under conditions that differ markedly from fully photosynthetic plants—specifically in lineages where plastid activity is developmentally partitioned and the plastomes themselves have undergone extensive structural changes.

Here, we report on the plastid editomes of four species in the order Schizaeales: two with achlorophyllous gametophytes—*Schizaea dichotoma* (L.) Sm. and *Actinostachys digitata* (L.) Wall. (Schizaeaceae)—and two with green, photosynthetic gametophytes—*Anemia phyllitidis* (L.) Sw. (Anemiaceae) and *Lygodium microphyllum* (Cav.) R. Br. (Lygodiaceae). We present newly assembled and annotated plastomes, validate, and characterize plastid RNA editing sites using transcriptome data, assess the abundance of PPR proteins, and apply phylogenetic and bioinformatic approaches to investigate how both C‐to‐U and U‐to‐C RNA editing evolves in relation to plastome structure and gametophyte photosynthetic capacity.

## RESULTS

### Plastome structure and content in Schizaeales

**Figure 1:**
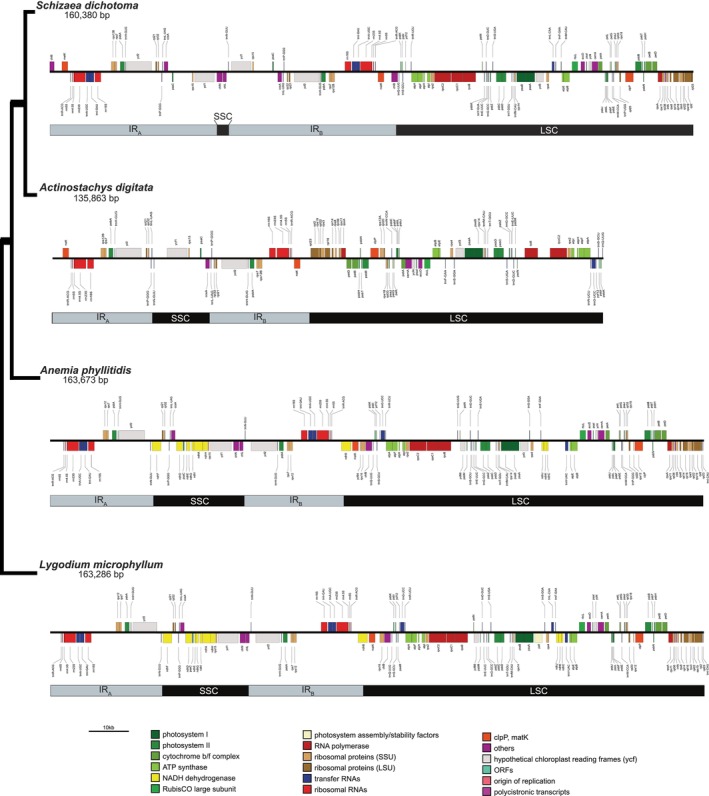
Linearized plastome maps for four Schizaeales species. Inverted repeat (IR) regions are outlined in gray and single‐copy regions (the small single copy [SSC] and large single copy [LSC]) in black. Phylogenetic relationships of the four species are shown on the left.

Our plastome assemblies from four Schizaeales species revealed remarkable diversity in both size and gene content. The plastomes of *A. phyllitidis* and *L. microphyllum* are similar in size, measuring 163 673 and 163 286 bp, respectively (Figure 1). By contrast, the plastomes of the two Schizaeaceae species are considerably smaller. *Schizaea dichotoma* has a plastome of 160 380 bp, while *A. digitata* has a notably reduced plastome at 135 863 bp (Figure 1).

In Schizaeaceae, we observed dramatic expansion of the IR regions accompanied by substantial reduction of the SSC region. *Lygodium microphyllum* and *A. phyllitidis* share a typical SSC structure, containing the same 14 protein‐coding genes, a portion of *ndhF*, and two tRNAs (Figure 1). In *A. digitata*, the IR has expanded slightly to incorporate *rps21* and *rps32*, which reside in the SSC in *L. microphyllum* and *A. phyllitidis* (Figure 1). IR expansion in *S. dichotoma* is even more extreme: its SSC is reduced to just 2863 bp and includes only *chlL*, *chlN*, and *trnN‐GUU* (Figure 1). This expansion brings *ccsA*, *psaC*, *rps15*, and *ycf1* into the IR—genes that remain in the SSC in the other three species (Figure 1). Additionally, *matK* has been translocated into the IR in both Schizaeaceae species, while it remains in the LSC region in *L. microphyllum* and *A. phyllitidis* (Figure 1).

**Figure 2:**
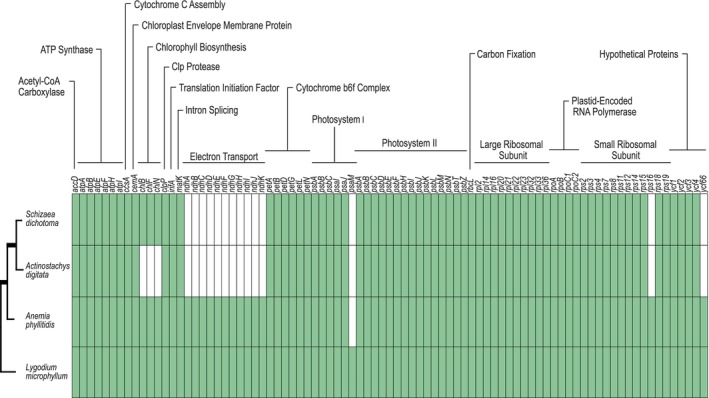
Gene presence/absence in four plastomes of Schizaeales. Phylogenetic relationships of the four species are shown on the left. Green cells represent the presence of a given gene while white cells represent absence. General functional groups are shown above the gene names.

Extensive plastid gene loss was observed in Schizaeaceae. Both *S. dichotoma* and *A. digitata* have lost the entire suite of 11 NAD(P)H dehydrogenase (*ndh*) genes for electron transport, as well as *rps16* and *ycf66* (Figure 2). In addition, *A. digitata* has lost all three genes associated with chlorophyll biosynthesis: *chlB*, *chlF*, and *chlN* (Figure 2). The photosystem I *psaM* gene is absent from all plastomes assembled here except for *L. microphyllum* (Figure 2). In total, we detected 86 protein‐coding genes in *L. microphyllum*, 85 in *A. phyllitidis*, 72 in *S. dichotoma*, and 69 in *A. digitata* (Figure 2). To determine whether the missing *ndh* genes might have been functionally transferred to the nucleus in Schizaeaceae, we searched the assembled transcriptomes of *A. digitata* and *S. dichotoma*, but found no evidence of these genes, suggesting that they have been entirely lost from Schizaeaceae. Likewise, the missing *chl* genes from *A. digitata*, were also entirely lost.

### Plastid RNA editing evolution

**Figure 3:**
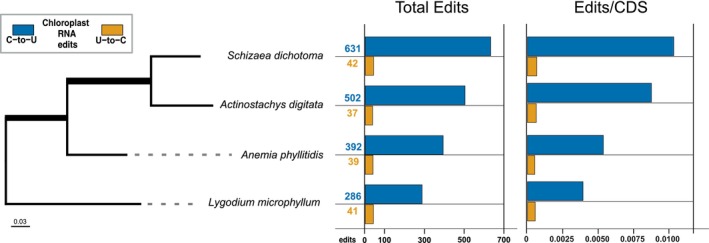
Chloroplast RNA editing levels in the Schizaeales. Phylogenetic relationships of the four species are shown on the left with branch lengths from a maximum likelihood analysis. Branch lengths are expressed in substitutions/site and a scale bar is in the bottom‐left corner. Blue horizontal bars show the number of C‐to‐U edits in the leftmost panel under ‘Total Edits’ and the number of C‐to‐U edits per the number of protein‐coding bases in the chloroplast genome in the rightmost panel under ‘Edits/CDS’. Similarly, gold bars indicate the number of total U‐to‐C edits or U‐to‐C edits per coding base on the left and right, respectively.

Analyses of chloroplast RNA editing in the Schizaeales revealed a sharp disparity between the two edit types. C‐to‐U editing levels were extremely variable ranging from as low as 286 C‐to‐U edits in *L. microphyllum* to 631 C‐to‐U edits in *S. dichotoma*, with both Schizaeaceae species displaying higher C‐to‐U editing levels than the non‐Schizaeaceae species (Figure 3). U‐to‐C editing levels, however, showed little variation ranging from 37 edits in *A. digitata* to 42 edits in *S. dichotoma* (Figure 3). The number of edits per protein‐coding base (CDS) in the plastomes was additionally calculated and mirrors the patterns of raw edit numbers in each lineage with the highest number of C‐to‐U edits per CDS in *S. dichotoma* and *A. digitata*, but little variation across the order for U‐to‐C edits (Figure 3).

**Figure 4:**
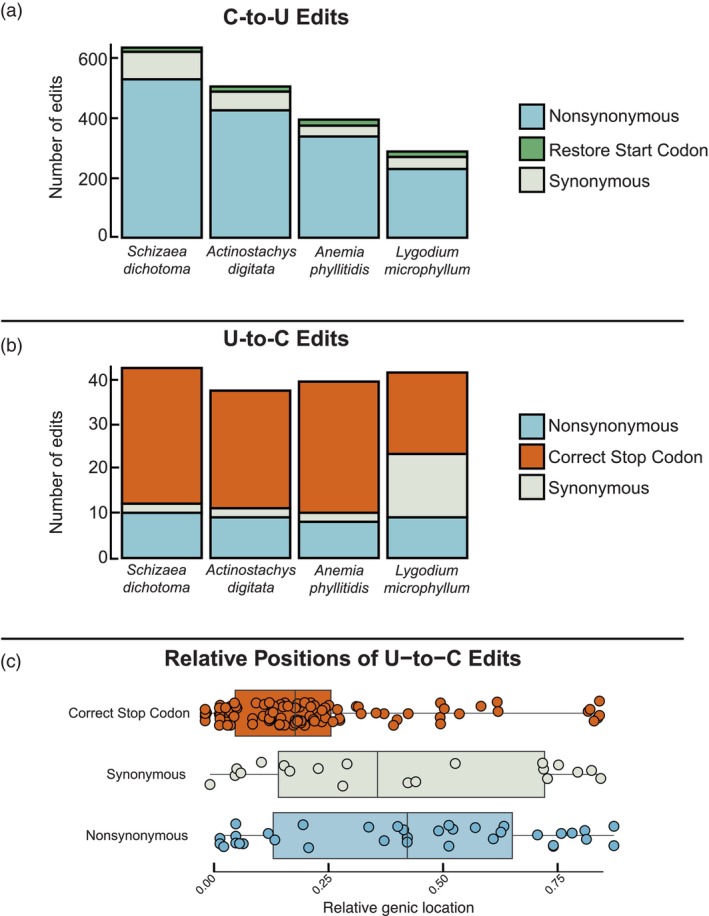
Proportion of RNA editing sites resulting in non‐synonymous (light blue), synonymous (gray) changes, as well as cryptic start codon restoration (green, C‐to‐U only) and internal stop codon correction (orange; U‐to‐C only). (a) C‐to‐U edits. (b) U‐to‐C edits. (c) Relative genic positions of U‐to‐C edits grouped by codon change type.

Most C‐to‐U edits in all four species resulted in non‐synonymous codon changes (Figure 4). A smaller proportion restored start codons by converting ACG to AUG, while others are synonymous and did not alter the amino acid sequence (Figure 4). By contrast, most U‐to‐C edits restored coding potential by converting internal stop codons to sense codons, while a few resulted in non‐synonymous amino acid substitutions (Figure 4). Like C‐to‐U edits, a small proportion are synonymous (Figure 4). Non‐synonymous and synonymous U‐to‐C edits tend to be evenly distributed across transcripts, while U‐to‐C edits that correct internal stop codons tend to be biased toward the 5′ end (Figure 4).

**Figure 5:**
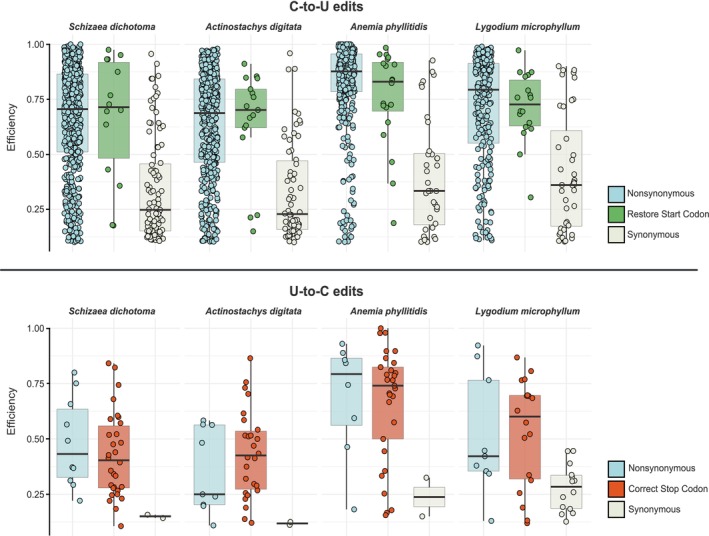
RNA efficiencies for four Schizaeales species. The top panel shows C‐to‐U editing efficiencies with non‐synonymous edits in light blue, synonymous edits in gray, and edits that restore start codons in green. The bottom panel shows U‐to‐C editing efficiencies with non‐synonymous edits in light blue, synonymous edits in gray, and edits that correct internal stop codons in orange.

RNA editing efficiencies—defined as the percentage of mapped RNA reads at a given site that are edited—were generally lower in the Schizaeaceae compared with *Lygodium* and *Anemia*, particularly for U‐to‐C edits (Figure 5). Across all species, synonymous edits were typically edited at low efficiency regardless of direction. Non‐synonymous C‐to‐U edits occurred at slightly higher efficiencies in *L. microphyllum* and *A. phyllitidis* than edits that restore cryptic start codons, whereas in *S. dichotoma* and *A. digitata*, their efficiencies were more comparable. U‐to‐C edits in the Schizaeaceae were often edited at less than 50% efficiency, contrasting with the higher efficiencies observed in *A. phyllitidis* and *L. microphyllum* (Figure 5).

**Figure 6:**
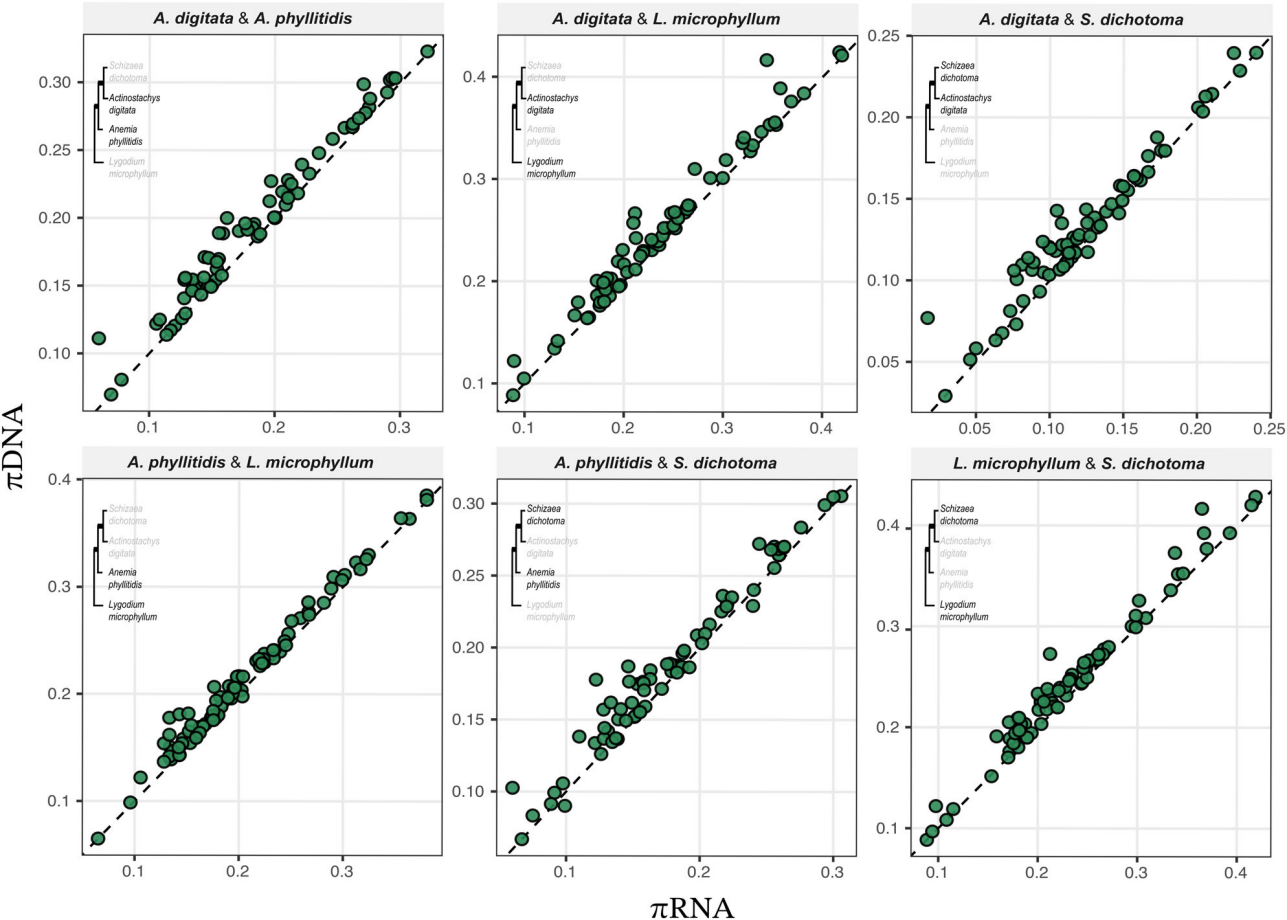
Pairwise nucleotide diversity (π) calculated on DNA alignments and post‐editing RNA alignments across six pairs of Schizaeales species for each plastid gene. DNA π is shown on the *y*‐axis and RNA π on the *x*‐axis. The dashed trendline represents the 1:1 line. Points above this line represent genes with a higher DNA π than RNA π and points below represent genes with a lower DNA π than RNA π.

**Figure 7:**
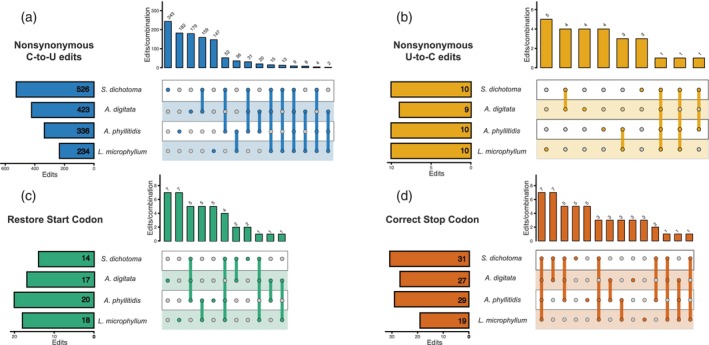
Proportion of shared RNA editing sites across Schizaeales. Non‐synonymous C‐to‐U edits are shown in blue (a), non‐synonymous U‐to‐C edits in yellow (b), C‐to‐U edits at start codons in green (c), and U‐to‐C edits at internal stop codons in dark orange (d). Horizontal bars to the left of species abbreviations show the number of edits present in each species. Vertical bars denote how many edits are shared by an exclusive group of taxa, defined by the connected dots.

Using gene alignments, we first compared nucleotide diversity (π) before and after editing and found that π from DNA alignments was consistently higher than π from post‐edited RNA alignments for nearly all genes and across all species pairs (Figure 6). We then assessed the conservation of individual edits and discovered that most non‐synonymous C‐to‐U and U‐to‐C edits were lineage specific (Figure 7). 40 genes across Schizaeales require C‐to‐U editing to restore the start codon in at least one species. Each species individually possesses between 14 and 20 start codon edits (Figure 7). These edits appeared slightly more conserved, with five start codon edits found in all species except *L. microphyllum* (Figure 7). We found no start codon edits to be conserved across all species. We recovered a total of 49 unique internal stop codons requiring correction by U‐to‐C editing across the Schizaeales, ranging from 31 in *S. dichotoma* to 19 in *L. microphyllum* (Figure 7). Of these, seven are conserved across the order, with another seven shared exclusively by the Schizaeaceae, and five more conserved between Schizaeaceae and *A. phyllitidis* (Figure 7).

**Figure 8:**
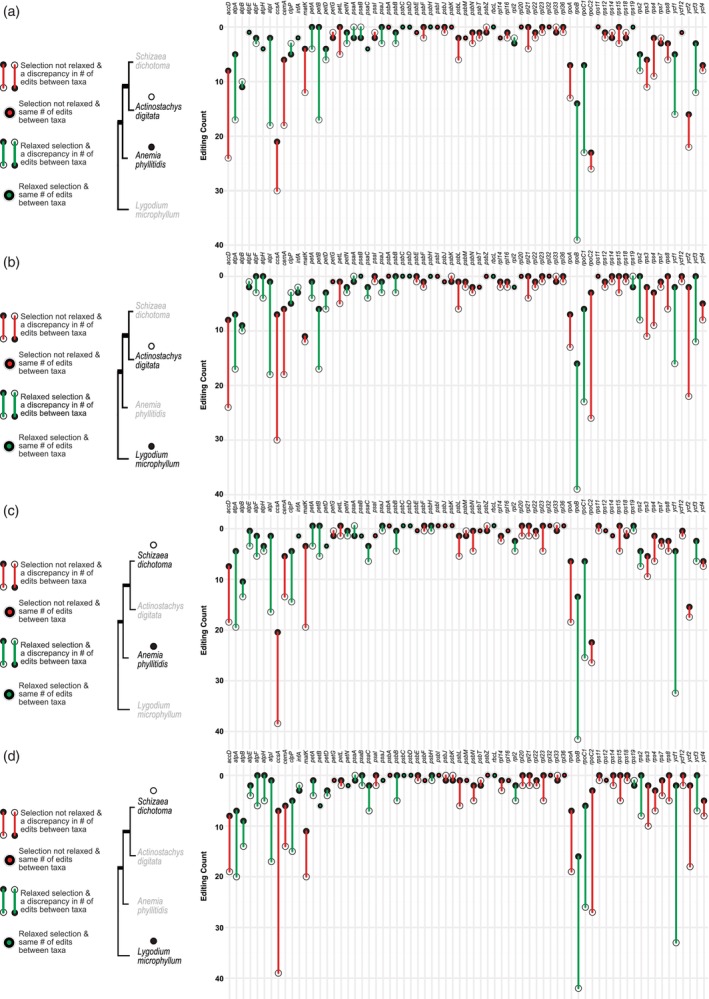
Pairwise differences in the number of C‐to‐U edits for each gene for every Schizaeaceae versus non‐Schizaeaceae comparison. Open circles are used for Schizaeaceae species (*Schizaea dichotoma* or *Actinostachys digitata*) and dark circles for non‐Schizaeaceae species (*Anemia phyllitidis* or *Lygodium microphyllum*). Genes under relaxed selection in the Schizaeaceae are shown in green, while genes not under relaxed selection are in red. (a) Pairwise comparison for *A. digitata* vs. *A. phyllitidis*. (b) Pairwise comparison for *A. digitata* vs. *L. microphyllum*. (c) Pairwise comparison for *S. dichotoma* vs. *A. phyllitidis*. (d) Pairwise comparison for *S. dichotoma* and *L. microphyllum*.

**Figure 9:**
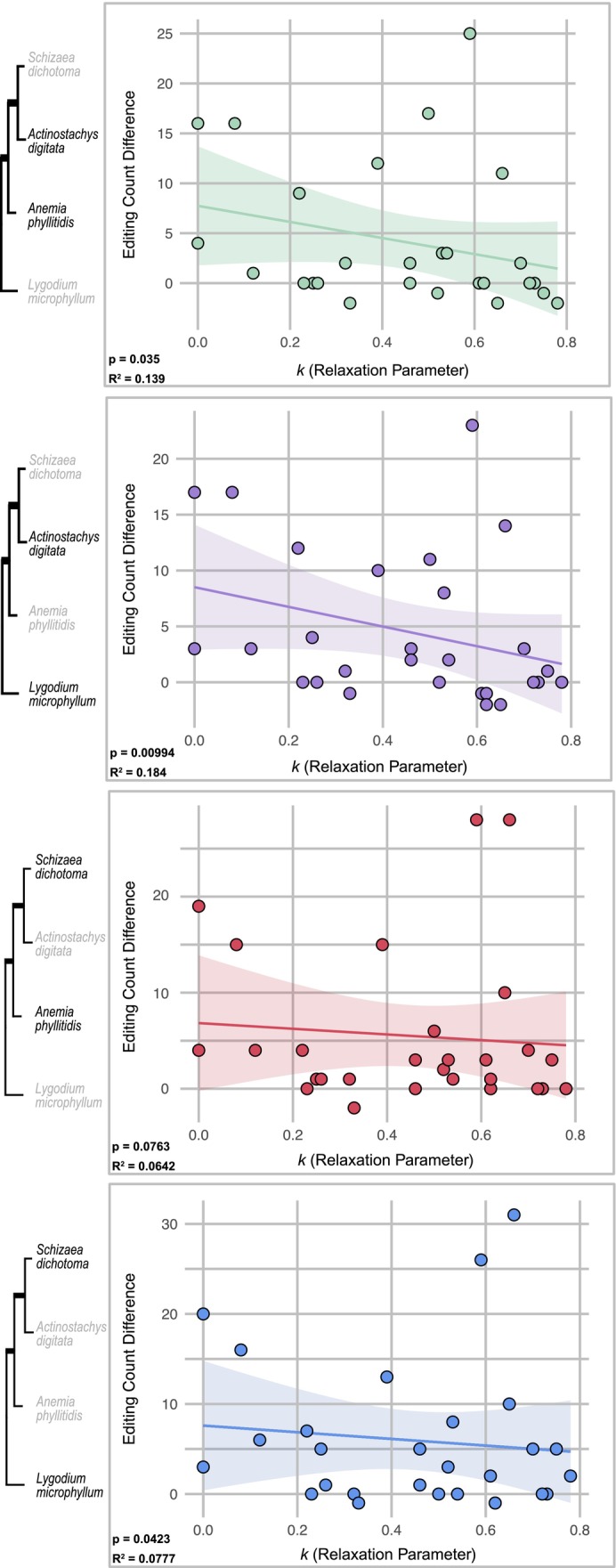
Relationship between relaxation parameter *k* and the pairwise difference in the number of C‐to‐U edits in every gene under relaxed selection. All Schizaeaceae versus non‐Schizaeaceae comparisons are shown.

Using a dataset with expanded sampling to include additional *Schizaea, Actinostachys*, and *Lygodium* species, we found statistically significant evidence for relaxed selection in the Schizaeaceae for 28 genes (Figure 8). Support for the relaxation of selection in Schizaeaceae was found in genes for nearly every functional group but was particularly pronounced for photosystem genes (*psa* and *psb*) as well as ATP synthase genes (*atp*; Figure 8). Plastid‐encoded RNA polymerase genes (*rpo*) and *ycf1* exhibited some of the highest pairwise non‐synonymous C‐to‐U editing differences between Schizaeaceae and non‐Schizaeaceae species, however, these are also among the longest genes in chloroplast genomes. To account for gene length, we ran a multiple linear regression to test the relationship between the relaxation parameter (*k*) and the pairwise editing difference between Schizaeaceae and non‐Schizaeaceae species in genes under relaxed selection, using gene length as a covariate. *k* represents the strength of selection relative to a reference set of branches—here the *Lygodium* and *Anemia* branches—and for genes where selection is relaxed, it ranges from 0 to approaching 1. *k* = 0 represents a complete relaxation of selection whereas values close to 1 represent genes where selection is only slightly relaxed. For the relaxed genes, *k* varies between 0 in some genes to 0.78. For three out of four comparisons, we find a significant negative relationship between *k* and the pairwise editing difference (Figure 9). The only comparison that did not meet a *P* < 0.05 significance threshold was *S. dichotoma* versus *A. phyllitidis*, however, the *P*‐value here is quite close at 0.0763 (Figure 9).

**Figure 10:**
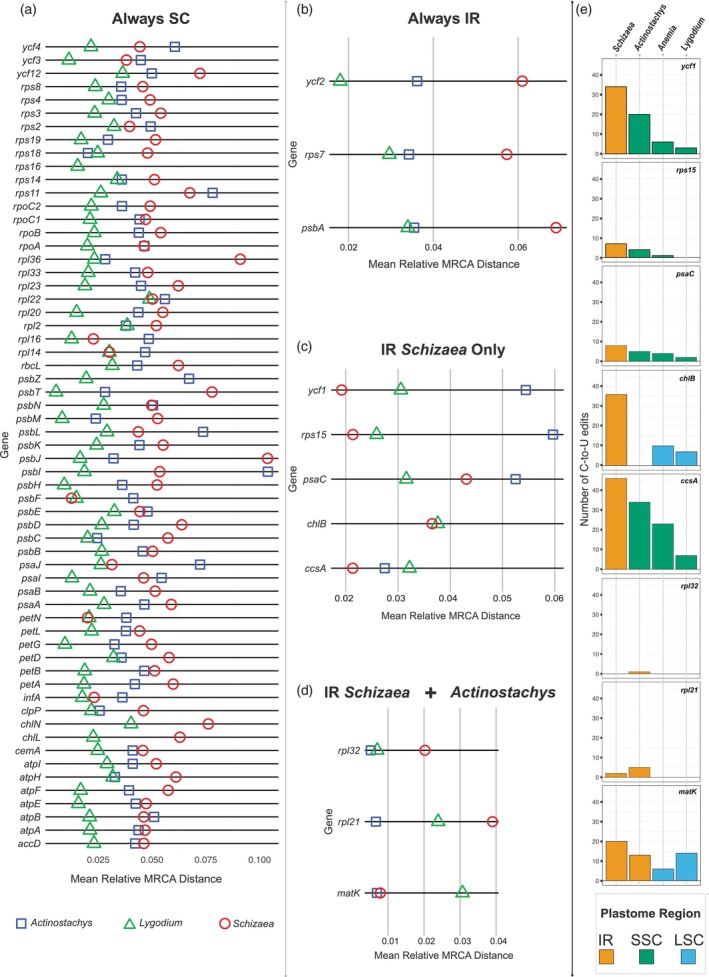
Comparisons of relative substitution rates for different plastid‐encoded genes across different Schizaeales species. Panels (a–d) show the mean relative distance to the most recent common ancestor (MRCA) for *Lygodium* (green triangle), *Actinostachys* (blue square), and *Schizaea* (red circle) for each plastid gene shared among the three genera. Panel (a) shows genes that are consistently found in the single‐copy regions, panel (b) shows genes found consistently in the inverted repeat (IR), (c) shows genes translocated into the IR in *Schizaea* only, and (d) shows genes translocated into the IR in *Schizaea* and *Actinostachys*. Panel (e) shows the amount of RNA editing for every gene that has been translocated for four Schizaeales species, colored by what region that gene resides in each species. IR is colored in gold, the small single‐copy region (SSC) in green, and the large single‐copy region (LSC) in blue.

For genes that are consistently either in single‐copy regions or the IR across Schizaeales, we found the mean relative MRCA distances to generally be lower in *Lygodium* than *Actinostachys* and *Schizaea* (Figure 10a,b). However, for the five genes that have been translocated into the IR in *Schizaea* specifically, *Schizaea* typically has a much lower mean relative MRCA distance than *Actinostachys* or *Lygodium*, with *psaC* being the only exception (Figure 10c). Three genes were translocated into the IR for both *Actinostachys* and *Schizaea*. Here, *Actinostachys* consistently has a lower mean relative MRCA distance than *Schizaea* and *Lygodium* (Figure 10d). For *matK*, *Schizaea* has a lower mean relative MRCA distance than *Lygodium*, very close to that of *Actinostachys*, but it has the highest mean relative MRCA distance for *rpl32* and *rpl21* (Figure 10d). For translocated genes, C‐to‐U RNA editing levels are consistently higher in species where that gene resides in the IR as opposed to the LSC or SSC (Figure 10e).

### Schizaeales PPR protein diversity

Using *de novo* assembled transcriptomes, we identified PPR genes in each of the four Schizaeales species. Using this method, we identified thousands of PLS‐class PPR proteins in each species (Table 1). However, we found very few of these to contain catalytic editing domains (DYW for C‐to‐U editing and DYW:KP for U‐to‐C editing; Table 1). At most, we found 35 full‐length C‐to‐U editing factors in *L. microphyllum* to as few as 4 in *S. dichotoma* (Table 1). Similarly, we found at most 16 full‐length U‐to‐C editing factors in *L. microphyllum* to as few as 4 in *A. digitata* (Table 1).

**Table 1:** Schizaeaceae pentatricopeptide repeat (PPR) proteins

	PLS	E+	E+:KP	DYW	DYW:KP	Total
*Schizaea dichotoma*	2304	18	16	4	6	2348
*Actinostachys digitata*	2182	13	16	6	4	2221
*Anemia phyllitidis*	3095	32	34	13	12	3186
*Lygodium microphyllum*	1266	280	214	35	16	1811

*Note*: PLS sequences are PPR proteins with PLS triplets, with a C‐terminal truncation with no distinctive features of C‐to‐U or U‐to‐C editing proteins. The E+ sequences are PPR proteins with PLS triplets, with a C‐terminal truncation ending in an E motif with signatures indicative of C‐to‐U editing proteins. The E+:KP sequences are PPR proteins with PLS triplets, with a C‐terminal truncation ending in an E motif with signatures indicative of U‐to‐C editing proteins. The DYW sequences are PPR proteins with a DYW domain. The DYW:KP sequences are PPR proteins with a DYW:KP domain.

## DISCUSSION

Over the past three decades, substantial progress has advanced our understanding of the evolutionary dynamics of RNA editing in plant organellar genomes (Small et al., 2020). A prevailing model proposes that editing sites originate through CNE, a ratchet‐like process driven by neutral mutations (Covello & Gray, 1993). However, the overall abundance of editing sites in a plastome is shaped by more than just their mode of origin. The high frequency of C‐to‐T mutations in AT‐rich plastid genomes (Huang et al., 2005) continuously erodes editing sites by converting editable cytidines into thymidines, thereby eliminating the need for editing. At the same time, not all potential editing sites are tolerated—selection may quickly purge sites that disrupt gene expression or impose burdens on highly transcribed genes. Thus, the observed distribution of RNA editing sites emerges from three competing forces: neutral origination via CNE, purifying selection that eliminates deleterious edits, and backmutation (e.g., C‐to‐T substitutions) that progressively erodes established sites (Fauskee et al., 2025; Ishibashi et al., 2019; Mower, 2008).

Despite these advances, plastid RNA editing evolution is well characterized in fully photosynthetic lineages but poorly understood in species with reduced or absent photosynthetic capacity. In these systems, plastid genomes often experience gene loss, and the selective constraints on retained plastid genes—particularly photosynthesis‐related genes—are presumably relaxed. This raises a key question: under relaxed selection, do editing sites accumulate more readily via CNE, or do gene losses and elevated mutation rates lead to a net reduction in editing sites? Addressing these questions is essential for understanding RNA editing evolution in functionally reduced plastid genomes.

Ferns are an excellent model for studying plastid RNA editing evolution. They retain higher C‐to‐U editing levels than seed plants and are among the few vascular plant lineages that retain U‐to‐C editing, which has been lost in seed plants. We investigated plastid RNA editing in the fern order Schizaeales, which comprises three families: Schizaeaceae, Anemiaceae, and Lygodiaceae (PPG I, 2016). While all species in Anemiaceae and Lygodiaceae exhibit typical photosynthesis in both gametophytes and sporophytes, Schizaeaceae includes several lineages with achlorophyllous, subterranean, mycoheterotrophic gametophytes (Bierhorst, 1975; reviewed in Ke et al., 2022). Here, we asked whether RNA editing levels differ between fully autotrophic and partially heterotrophic ferns, and what evolutionary forces might drive those differences.

Our plastome assemblies and annotations corroborate findings initially reported by Labiak and Karol (2017) as well as by Ke et al. (2022). Specifically, we recovered the same IR expansion and plastome reduction in *A. digitata* as well as the more extreme IR expansion and dramatic SSC reduction in *S. dichotoma* (Figure 1). We also recovered the same gene losses in the two Schizaeaceae species as previously reported, including the entire loss of the electron transport (*ndh*) gene suite, *psaM, rps16*, and *ycf66*, along with the specific loss of the three chlorophyll biosynthesis (*chl*) genes in *A. digitata* (Figure 2). Despite these extensive gene losses across Schizaeaceae, both species showed a dramatic increase in C‐to‐U RNA editing levels. The *S. dichotoma* plastome harbored more than double the number of C‐to‐U edits found in *L. microphyllum*, despite encoding 14 fewer protein‐coding genes (Figure 3). In contrast, there was strikingly little variation in the number of U‐to‐C edits across all species examined (Figure 3), supporting the hypothesis that C‐to‐U and U‐to‐C editing are likely shaped by different evolutionary pressures (Fauskee et al., 2021, 2025).

Consistent with other fern lineages (Fauskee et al., 2025), most C‐to‐U edits caused non‐synonymous amino acid substitutions, with some restoring start codons, whereas U‐to‐C edits primarily corrected internal stop codons (Figure 4). Both Schizaeaceae species exhibited lower C‐to‐U RNA editing efficiencies than *Anemia* and *Lygodium*, across all edit types: non‐synonymous edits, synonymous edits, and edits restoring start codons (Figure 5). Schizaeaceae also exhibited an even more dramatic reduction in U‐to‐C editing efficiency relative to *Anemia* and *Lygodium* (Figure 5). In Schizaeaceae, most U‐to‐C edits are edited at below 50% efficiency, whereas many U‐to‐C edits in *Anemia* and *Lygodium* have much higher efficiencies (Figure 5). Across the Schizaeales, RNA editing efficiencies at internal stop codons are quite variable, biased toward the 5′ end of the transcript, and appear to be evolutionarily conserved (Figures 4 and 5). These observations are consistent with the hypothesis that selective U‐to‐C editing of internal stop codons functions in plastid gene regulation in ferns (Fauskee et al., 2025; Kwok van der Giezen et al., 2025).

Schizaeaceae exhibited elevated C‐to‐U RNA editing site numbers relative to *Anemia* and *Lygodium* (Figure 3), but reduced editing efficiencies (Figure 5). We asked whether the increased number of edits reflects a greater accumulation and retention of true editing sites (through higher rates of site gain and/or lower rates of site loss), or ‘promiscuous’ off‐target editing by PPR proteins at mismatched binding sites. Off‐target edits would likely show low efficiency because of imperfect binding site matches. To assess these alternatives, we calculated pairwise nucleotide diversity (π) for each gene and compared π calculated from DNA alignments to π from post‐edited RNA alignments (Figure 6). If the increased editing reflects off‐target activity, then the post‐edited RNA should show higher π than the genomic DNA, as these non‐corrective edits would introduce additional sequence variation in the mRNA relative to the DNA. Alternatively, if the higher edit numbers stem from a higher rate of gaining edits through CNE and/or a decreased rate of losing edits through backmutation, we would expect to see higher DNA π than RNA π. This is because edits acquired through CNE are predominantly corrective so differences in the DNA will be resolved in the RNA via RNA editing. Across nearly all genes and pairwise comparisons, DNA π exceeded RNA π (Figure 6), indicating limited off‐target editing. Instead, the difference in the amount of RNA editing is likely due to some combination of increased rates of editing site establishment through CNE and decreased rate of losing edits through backmutation. Currently, we lack the sampling to distinguish between the two possibilities, though it is likely both play a significant role in the overall editing distribution.

The overwhelming majority of plastid RNA editing events in Schizaeales are non‐synonymous C‐to‐U edits (Figure 4). These edits are not evolutionarily conserved—each species harbors over 100 unique editing sites not shared with any other species (Figure 7). Our selection analyses show that 28 genes are under relaxed selection in Schizaeaceae relative to *Anemia* and *Lygodium* (Figure 8). Many of the largest pairwise differences in the number of non‐synonymous C‐to‐U edits—comparing Schizaeaceae to *Anemia* and *Lygodium*—occur in these relaxed genes, particularly *ycf1*, *rpoB*, and *rpoC1* (Figure 8). Furthermore, in all but one pairwise comparison (*Schizaea* versus *Anemia*), we find a statistically significant negative relationship between editing count differences and *k*, suggesting that genes under relaxed selection accumulated more non‐synonymous C‐to‐U edits in Schizaeaceae than in their photosynthetic relatives (Figure 9).

This result is somewhat unexpected. Previous studies in both ferns (Fauskee et al., 2025) and angiosperms (Ishibashi et al., 2019; Mower, 2008) report a consistent trend of C‐to‐U editing site loss over time, with faster evolving lineages typically harboring fewer such edits. The elevated substitution rates in Schizaeaceae (Figure 3) suggest accelerated backmutation, which would be expected to reduce editing levels. Instead, we observe the opposite: Schizaeaceae species possess a higher number of C‐to‐U edits. This pattern suggests that relaxed selection facilitates the establishment of new editing sites. Supporting this interpretation, genes under weaker selection tend to accumulate more edits in Schizaeaceae compared with their orthologs in *Anemia* and *Lygodium* (Figure 9), and the DNA–RNA π comparison confirms that the overall editing difference is indeed due to novel unique edits, not off‐target editing (Figure 6). Taken together, these results point to a balance between selection and the neutral origin of edits via CNE. When selection is relaxed, newly arising editing sites are more likely to persist, and their accumulation scales with the degree of relaxation (Figure 9). Moreover, PLS‐class PPR proteins are known to strongly prefer binding to uridines (Dennis et al., 2025). The high rate of C‐to‐T substitutions in plastid genomes—especially in genes under relaxed selection—may generate additional U‐rich motifs that PPR proteins can recognize, thereby expanding the pool of potential editing sites. Thus, the elevated number of C‐to‐U edits in Schizaeaceae likely reflects an accelerated CNE ratchet, wherein relaxed selection simultaneously increases mutation rate, increases the availability of editable U‐rich motifs, and permits the persistence of editing sites that would be eliminated under stronger constraint. In other words, relaxed selection both increases the number of potential PPR binding sites through C‐to‐T substitutions that generate U‐rich motifs—and reduces barriers to retaining established edits, together promoting the extraordinary accumulation of editing sites in Schizaeaceae.

Beyond relaxed selection, a second factor contributing to elevated RNA editing levels in Schizaeaceae is expansion of the IR. Using our expanded sampling, we calculated the mean branch length from each *Lygodium*, *Actinostachys*, and *Schizaea* species to the most recent common ancestor (MRCA) of their respective genus. For genes consistently located in either the IR or the single‐copy regions throughout their evolution, *Schizaea* and *Actinostachys* tend to have relatively longer branches than *Lygodium* (Figure 10a,b). However, genes translocated into the IR showed the opposite pattern. In both *Schizaea* and *Actinostachys*, IR‐relocated genes generally showed shorter relative mean branch lengths than the same genes in *Lygodium* (Figure 10c,d), suggesting reduced substitution rates following IR integration. This agrees with previous findings that IR‐relocated genes experience reduced substitution rates in ferns (Li et al., 2016). Consequently, for every case of IR translocation, C‐to‐U editing levels were consistently higher in the species harboring the gene in the IR (Figure 10e). These results suggest that the reduced substitution rates associated with IR translocation slow the erosion of C‐to‐U editing sites. In effect, the IR ‘protects’ editing sites from being lost over time, because the lower mutation rate reduces the chance of backmutation at edited sites.

Interestingly, a different pattern is observed in the lycophyte *Phylloglossum drummondii*, which has a partially subterranean and tuberous gametophyte stage. In contrast with the Schizaeaceae, *P. drummondii* retains most of the conserved plastid genes, including the often‐lost *ndh* suite, and exhibits relatively low levels of RNA editing (Kwok van der Giezen et al., 2024). Although interpretation is complicated by a generally low number of plastid RNA editing sites in huperzioid lycophytes overall, *P. drummondii* provides a notable exception whereby the transition to a partially non‐photosynthetic lifestyle is not accompanied by plastid genome degradation or elevated RNA editing.

C‐to‐U and U‐to‐C RNA editing in plant organelles are carried out by PPR proteins that either possess or interact with a cytidine deaminase‐like C‐terminal domain called the DYW or DYW:KP domain, respectively. In angiosperms, PPR proteins interact with several auxiliary factors such as RIP/MORF, ORRM, and OZ1 proteins (Li et al., 2025). However, these are absent in seed‐free plants where PLS motif tracts tend to be longer and may act independently. An unexpected finding was the striking lack of full‐length PPR–RNA editing factors in Schizaeales (Table 1). Across Schizaeales, we recovered far fewer PPR proteins with C‐terminal editing domains than would be expected given the number of plastid RNA editing sites. This shortfall is even greater when taking into account the extensive RNA editing known to occur in the mitochondrial genomes of several fern species (Guo et al., 2017; Knie et al., 2016; Zumkeller et al., 2023). However, we did recover a large number of PLS‐class PPR proteins lacking their catalytic C‐terminal domains which could account for the high number of editing sites found in both the plastid and presumably mitochondrial genomes. These PLS proteins contain the RNA‐binding arrays responsible for recognizing editing sites by binding to upstream *cis* sequences. This strongly suggests that in Schizaeales—and likely in ferns more broadly (Gutmann et al., 2020; Li et al., 2018)—the RNA binding and catalytic functions of RNA editing are carried out by separate proteins assembled *in trans*, as is the case for some angiosperm PPR proteins such as Arabidopsis CRR4 which interacts with DYW1 to edit the plastid ndhD‐1 editing site (Boussardon et al., 2012). In the distantly related Salviniales ferns, the number of DYW and DYW:KP domains responsible for C‐to‐U and U‐to‐C conversion, respectively, is far outnumbered by the abundance of RNA editing sites and PLS PPR proteins (Li et al. 2018). This mismatch in Schizaeales is even more striking, where as few as 4 DYW domains appear to serve 631 C‐to‐U editing sites in the *S. dichotoma* chloroplast transcriptome as well as all the edits in the mitochondrial transcriptome. Some seed‐free lineages, such as the hornwort *Anthoceros*, have a near one‐to‐one ratio of full‐length U‐to‐C editing factors, but a mismatch in the number of DYW domains to C‐to‐U editing sites (Gutmann et al., 2020). We cannot exclude the possibility of an unidentified seed‐free plant‐specific RNA editing cofactor protein or protein complex, but it seems apparent that DYW and DYW:KP domains can act as ‘donor’ catalytic domains for both C‐to‐U and U‐to‐C editing, and this may be the dominant mode of plastid and mitochondrial editing proteins in ferns.

The lack of protocols for genetic manipulation of a fern model organism makes characterization of specific RNA editing proteins impossible and although this has been achieved in heterologous expression systems previously (Lesch et al., 2022; Oldenkott et al., 2019; Royan et al., 2021), the redundancy of the PPR–RNA recognition code (Barkan et al., 2012) makes identification of specific PPR protein editing sites unviable because a single PPR protein may be predicted to bind many RNAs. However, Schizaeales ferns are a promising model for studying the protein–protein interface between PLS PPR proteins and donor DYW/DYW:KP domains due to the small number of DYW/DYW:KP genes. Decoupling of the PLS RNA‐binding domain from a fixed catalytic identity (e.g., C‐to‐U versus U‐to‐C) may allow for greater flexibility in site targeting which may influence the rapid accumulation of RNA editing sites. This modularity may help explain why fern plastomes harbor far more editing sites than those of angiosperms.

Together, our findings provide new insight into the evolutionary dynamics that shape plastid RNA editing in ferns and, more broadly, in land plants. Previous studies have emphasized the gradual erosion of editing sites over time, primarily driven by high C‐to‐T mutation rates that eliminate sites through backmutation, and selection can further prevent the establishment of editing sites in highly expressed genes where the additional complexity of RNA editing may be less tolerable. By examining species with non‐photosynthetic gametophytes, we reveal two distinct mechanisms that together explain how Schizaeaceae accumulate more C‐to‐U editing events than their fully photosynthetic relatives, despite possessing fewer protein‐coding genes and having higher overall substitution rates. The first is structural: expansion of the IR in *Schizaea* and *Actinostachys* decelerates substitution rates in translocated genes, reducing the frequency of backmutation at edited cytidines and thereby preserving editing sites that would otherwise be eroded. This result is entirely consistent with the established expectation that mutation rate and editing site retention are inversely related. The second mechanism is less intuitive. In single‐copy regions, substitution rates are elevated, which by the same logic should accelerate editing site loss. Yet, we observe the opposite. We argue that three interacting consequences of relaxed selection together resolve this paradox. First, overall mutation rates are elevated across the board, meaning T‐to‐C mutations—which generate new editing substrates—also occur more frequently. Second, the accompanying increase in C‐to‐T substitutions expands the pool of U‐rich sequence context that PPR editing factors can recognize, creating more potential *cis*‐binding domains and broadening the targeting repertoire of existing editing machinery. Third, and most importantly, when purifying selection on plastid gene function is weakened, the mutations that generate new editing sites are no longer efficiently purged—allowing newly arising sites to establish and persist where they previously could not. The net result is that editing site gain outpaces erosion even against an elevated mutational background. Finally, we find that Schizaeales plastomes contain far more editing sites than can be explained by the number of full‐length PPR editing factors alone. Instead, these species harbor many PLS‐class PPR proteins that lack catalytic domains, suggesting that RNA binding and catalytic functions are modular and assembled *in trans*, potentially allowing a single catalytic component to serve multiple editing complexes and facilitating the rapid accumulation and retention of editing sites. Altogether, our results highlight how relaxed selection and plastome structural change can expand plastid editomes through mechanisms that appear contradictory but are in fact complementary—one preserving editing sites by slowing the mutational clock, the other accumulating them by uncoupling mutation rate from selective constraint—and how examining partially non‐photosynthetic systems illuminates the interplay between neutrality, selection, and molecular machinery in shaping RNA editing evolution.

## MATERIALS AND METHODS

### Sampling, DNA extraction, and sequencing, plastome assembly, and annotation

DNA was extracted from four species in three families in the order Schizaeales: *S. dichotoma* and *A. digitata* (Schizaeaceae), *A. phyllitidis* (Anemiaceae), and *L. microphyllum* (Lygodiaceae). DNA was extracted from green, photosynthetic sporophyte leaf tissue and DNA isolations were performed using the E.Z.N.A. SP Plant and Fungal DNA Extraction Kit from Omega Bio‐Tek (Omega Bio‐Tek, Norcross, GA, USA; D5511). Isolated DNA was then quantified using a Qubit 2 Fluorometer (Thermo Fisher Scientific Inc., Walden, MA, USA) and the Qubit dsDNA High Sensitivity Quantification Assay kit (Thermo Fisher Scientific Inc., Walden, MA, USA; Q32851). DNA libraries for Illumina sequencing were then constructed using the NEBNext Ultra II DNA Library Prep Kit for Illumina (New England Biosciences, Ipswitch, MA, USA; E7645) following the manufacturer's default protocol for 200 base‐pair inserts. To enable multiplexing, each sample was tagged with a unique barcode using the NEBNext Multiplex Oligos for Illumina (New England Biosciences, Ipswitch, MA, USA; E6609). Resulting DNA libraries were sequenced on the Illumina Novaseq X Plus by Novogene Co., Ltd (Beijing, China) using 150 base‐pair, paired‐end chemistry. Demultiplexing was also performed by Novogene Co., Ltd.

Paired‐end DNA reads were uploaded to the Duke Compute Cluster (Duke University, Durham, NC, USA) where adapters were trimmed and low‐quality reads were removed using Trimmomatic version 0.39 (Bolger et al., 2014) with the following settings: LEADING:3 TRAILING:3 SLIDINGWINDOW:4:15 MINLEN:36. Using the resulting trimmed and paired reads, chloroplast genomes were assembled *de novo* using NOVOPlasty version 4.3.3 (Dierckxsens et al., 2017). For each assembly, an *rbcL* sequence was obtained from previously published full plastomes uploaded in GenBank for the same species (but different voucher) and used as the seed sequence. Specifically, ON207052 for *S. dichotoma*, ON207049 for *A. digitata*, OM990738 for *A. phyllitidis*, and MG761729 for *L. microphyllum*. Draft plastomes were then polished iteratively using Pilon (Walker et al., 2014) until Pilon suggested no further changes to the assembly. Polished plastome assemblies were then annotated in Geneious version 2022.0.2 using the BLAST plugin followed by manual adjustment. The same Genbank vouchers from which seed sequences were obtained were used as the annotation reference. Plastome assemblies were uploaded to Genbank with accession numbers available in Appendix S1. Annotated platomes are also available as Genbank files on GitHub at https://github.com/bfauskee/fauskee‐fern‐rna‐editing‐scripts/tree/main/assemblies/schizaeales.

### Transcriptome sequencing and assembly

RNA was extracted from the same vouchers for which plastome assemblies were generated. The RNA was extracted from either fresh, flash‐frozen, or RNApreserve‐fixed (BIONOVAS, Toronto, Canada) green sporophyte tissue. The RNA extractions were performed using the E.Z.N.A. Plant RNA Kit from Omega Bio‐Tek (Omega Bio‐Tek, Norcross, GA, USA; R6827), or CTAB‐column (RNA spin columns from Spectrum Total Plant RNA Kit; Millipore Sigma, Darmstadt, Germany) approach (Pelosi et al. 2024). During RNA extraction, samples were additionally treated with DNase I to reduce DNA contamination. DNase I treatments were performed with the Millipore Sigma DNase I kit (Millipore Sigma, Darmstadt, Germany; 69182). RNA extractions were quantified using the Qubit 2 Flurometer (Thermo Fisher Scientific Inc., Walden, MA, USA) and the Qubit RNA High Sensitivity Quantification Assay kit (Thermo Fisher Inc., Walden, MA, USA; Q32852).

RNA (cDNA) libraries were constructed using the Zymo‐Seq RiboFree Total RNA Library Kit (Zymo Research, Irvine, CA, USA; R3000) or the NEBNext Ultra II Library Prep (New England Biosciences, Ipswich, MA, USA; E7775), with ribosomal depletion probes designed for plant samples supplied by New England Biosciences as part of a beta test agreement. For both kits, library preparation was performed following suggested manufacturer's protocols. Unique barcodes were ligated to each sample to enable multiplexing, and the resulting libraries were sequenced by Novogene Co., Ltd (Beijing, China) and Genomics Bio Sci. & Tech. (New Taipei City, Taiwan) using 150 base‐pair, paired‐end chemistry on the Illumina NovaSeq X Plus or HiSeq X 10. All data quality control and demultiplexing were carried out by Novogene and Genomics Bio Sci. & Tech. Raw transcriptomic and genomic data generated for this project are available from the Sequence Read Archive (SRA), BioProject number: PRJNA1374273 and PRJNA1291224. Specific SRA accession numbers are available in Appendix S1.

### *De novo* transcriptome assembly

Raw transcriptomic data were trimmed of adapters using BBduk from the BBmap suite (Bushnell 2014). Trimmed reads were assembled using rnaSPAdes v4.0 with in assembler‐only mode (Bushmanova et al., 2019). The completeness of the resulting transcriptome assemblies was assessed with benchmarking using single‐copy orthologues, referencing the embryophyta_odb10 dataset (Simão et al., 2015).

### Identification of PPR protein sequences

Transcriptomes for each species were scanned for open reading frames (ORFs) in all six (three forward, three reverse) reading frames using the Julia script orfinder.jl (Kwok van der Giezen et al., 2025) and translated to a protein FASTA file. Models of PPR proteins were identified in the ORFs using ‘hmmsearch’ (HMMER v3.2.1) and the ‘DYW/DYW:KP’ hidden Markov model for PPR proteins described in Gutmann et al. (2020). PPRFinder (Gutmann et al., 2020) was run on the hmmsearch output to generate files containing annotated PPR motif and domain structures.

### RNA editing detection

To detect plastid RNA editing sites in the four Schizaeales plastomes, all protein‐coding gene sequences for each species were extracted using Geneious (Kearse et al., 2012) along with 100 base‐pairs flanking the beginning and end of each gene. For each species, protein‐coding genes along with flanking regions were combined into a single multi‐fasta file. A custom RNA editing detection pipeline modified from Edera and Sanchez‐Puerta (2021) was implemented to detect potential RNA editing sites. In this pipeline, RNA reads are trimmed twice with Trimmomatic (version 0.39), first in paired‐end mode using the following settings: LEADING:3 TRAILING:3 SLIDINGWINDOW:4:15 MINLEN:36 to remove adapters and low‐quality reads, then again in single‐end mode using the HEADCROP:13 setting which removes the first 13 bases in each read where GC content was non‐uniform. RNA reads were then mapped to the plastid protein‐coding genes for each species using Bowtie2 version 2.2.4 (Langmead & Salzburg, 2012). A BAM file was then generated using Samtools version 1.14 (Li et al., 2009). The total number of RNA reads mapped to each site in each gene, as well as the number of reads with each nucleotide present at that site, were calculated using bam‐readcount version 0.8.0 (Khanna et al., 2022) and custom Linux commands. This workflow ultimately output a TSV file showing the base present in the DNA at every site in every gene analyzed, the total number of mapped RNA reads to each site, and the number of mapped reads displaying each of the four nucleotides. An example script for these steps is available on GitHub (https://github.com/bfauskee/fauskee‐fern‐rna‐editing‐scripts).

Using the R pipeline described in Fauskee et al. (2025), putative RNA editing sites were detected and characterized. An example of this R pipeline is available on GitHub (https://github.com/bfauskee/fauskee‐fern‐rna‐editing‐scripts). Sites were considered RNA editing sites if they met the following conditions: at least 10 RNA reads mapped to the site and at least three reads and 10% of the total mapped RNA reads had the edited base (for example, a T mapped to a C for a C‐to‐U editing site). Uncommon regions with very low RNA read coverage were manually inspected. This pipeline also characterizes the amino acid change induced by each RNA editing event, the codon position it occurs at, the efficiency of each editing site, along with several other features. RNA editing efficiency is defined as the proportion of mapped RNA reads displaying the edited base (e.g., the number of reads with a T mapped to a C divided by the total number of reads mapped to that site).

### Evolutionary analyses of RNA editing sites

To understand how RNA editing sites are evolving in the Schizaeales, we performed several evolutionary and phylogenetic analyses. First, we aligned the coding regions of all plastid protein‐coding genes for each of the four Schizaeales species using MAFFT version 7.505 (Katoh & Standley, 2013). We then used the alignment position of each site to compare the presence and absence of each unique RNA editing site across the four species by generating UpSet plots using the R package UpSetR version 1.4 (Conway et al., 2017).

Next, we aligned the coding regions of each plastid protein‐coding gene again using MAFFT version 7.505 (Katoh & Standley, 2013), but with the addition of the previously published plastome of *Salvinia cucullata* (Genbank accession number: MF177095.1). Genes were concatenated and partitioned by gene using AMAS (Borowiec, 2016) to generate a supermatrix. We then carried out maximum likelihood (ML) tree estimation on the concatenated plastid supermatrix in IQ‐TREE2 version 2.2.2.7 (Minh et al., 2020) using 1000 ultrafast bootstrap replicates. Substitution model selection and optimal partitioning schemes were automated in IQ‐TREE2 using the MFP + MERGE option. For substitution model selection, the model preferred by the Bayesian information criterion (BIC) for each partition model was used. The concatenated ML tree was then rooted with *S. cucullata*, which was then pruned from the tree using the Phyx package version 1.3 (Brown et al., 2017).

We also calculated nucleotide diversity (π) to test whether differences in RNA editing among species reflect off‐target edits. Using the Schizaeales‐only multiple sequence alignments described previously, we created additional ‘post‐edited’ alignments by recoding RNA editing sites as the post‐edited base (changing a C to a T for a C‐to‐U edit and a T to a C for a U‐to‐C edit) without re‐aligning the sequences. These ‘post‐edited’ alignments represent the mRNA sequences. We then calculated pairwise π for all genes across all six species combinations on both the unedited DNA alignments and the recoded RNA alignments using custom R scripts. Lastly, we plotted DNA π versus RNA π.

To assess whether selection is relaxed in any plastid genes in Schizaeaceae relative to Anemiaceae and Lygodiaceae, we expanded our dataset to include several additional Schizaeales plastomes from Genbank. These included: *Schizaea elegans* (KX258660), *Schizaea pectinata* (NC035808), *Actinostachys pennula* (KU764518), *Lygodium flexuosum* (OM350009), *Lygodium merrillii* (OM350010), *Lygodium japonicum* (KC356645), and *Lygodium circinatum* (OM327797). Using the same alignment, concatenation, and phylogenetic inference methods outlined above, we constructed another phylogenetic tree for our expanded dataset. This resulting ML tree was similarly rooted with *S. cucullata* and then *S. cucullata* was pruned. We then assessed whether selection was relaxed in any plastid protein‐coding genes in Schizaeaceae relative to Anemiaceae and Lygodiaceae. Here only genes present across all species were analyzed, resulting in 69 protein‐coding genes. The selection analysis was carried out using RELAX (Wertheim et al., 2015) implemented in HYPHY version 2.5.64 (Kosakovsky Pond et al., 2020). For genes with relaxed selection (*k* value significantly less than 1), we compared the *k* value (lower values representing more extreme relaxation of selection) to the pairwise difference in the number of non‐synonymous C‐to‐U edits between each Schizaeaceae and non‐Schizaeaceae species. This yielded four pairwise comparisons: *S. dichotoma* versus *A. phyllitidis*, *S. dichotoma* versus *L. microphyllum, A. digitata* versus *A. phyllitidis*, and *A. digitata* versus *L. microphyllum*. For each pairwise comparison we plotted the pairwise C‐to‐U editing count difference by the k value for genes that had a *k* value significantly less than 1 (*P* < 0.05). We performed a multiple linear regression to quantify the relationship between *k* and the pairwise editing difference with gene length as a covariate in R (R Core Team 2021).

We also investigated whether genes translocated into the IR in the Schizaeaceae exhibited decelerated substitution rates, as has been observed in other fern lineages (Li et al., 2016). We constructed individual gene trees for each chloroplast gene using our expanded sampling dataset outlined above. The gene trees were built in IQ‐TREE2 (Minh et al., 2020) with substitution model selection carried out by using the MFP option. Again, the substitution model preferred by BIC was used. Each gene tree was then rooted with *S. cucullata* and then *S. cucullata* was pruned. For each gene tree, we calculated the mean relative distance to the MRCA for *Schizaea*, *Actinostachys*, and *Lygodium*. *Anemia* was excluded here because we only had access to one *Anemia* species. The mean relative distance to the MRCA for each genus was calculated by taking the mean branch length distance to the MRCA for each *Schizaea, Actinostachys*, or *Lygodium* species and dividing that mean by the total tree length. This enabled meaningful comparisons across genes. Distances were extracted from trees using the distRoot function in the adephylo R package version 1.16 (Jombart et al., 2010). We then binned genes by whether they were found consistently in single‐copy regions (SSC or LSC), consistently found in the IR, found in the IR in both *Actinostachys* and *Schizaea*, or found in the IR only in *Schizaea*. We then plotted the mean relative MRCA distance for each of the three genera for each gene.

## CONFLICT OF INTEREST

None of the authors have a conflict of interest to disclose.

## Supporting information


**Appendix S1.** Voucher information and data accession numbers for species studied here. Accession numbers for raw genomic and transcriptomic reads are available along with accession numbers for chloroplast genome assemblies.

## Data Availability

All data generated in this project are publicly available with details provided in Appendix S1. Bioinformatic code is additionally available at the GitHub links in this manuscript.

## References

[tpj70919-bib-0001] Barkan, A. , Rojas, M. , Fujii, S. , Yap, A. , Chong, Y.S. , Bond, C.S. et al. (2012) A combinatorial amino acid code for RNA recognition by pentatricopeptide repeat proteins. PLoS Genetics, 8, e1002910. 22916040 10.1371/journal.pgen.1002910 PMC3420917

[tpj70919-bib-0002] Barrett, C.F. , Freudenstein, J.V. , Li, J. , Mayfield‐Jones, D.R. , Perez, L. , Pires, J.C. et al. (2014) Investigating the path of plastid genome degradation in an early‐transitional clade of heterotrophic orchids and implications for heterotrophic angiosperms. Molecular Biology and Evolution, 31, 3095–3112. 25172958 10.1093/molbev/msu252

[tpj70919-bib-0003] Bellot, S. & Renner, S.S. (2016) The plastomes of two species in the endoparasite genus *Pilostyles* (Apodanthaceae) each retain just five or six possibly functional genes. Genome Biology and Evolution, 8, 189–201. 10.1093/gbe/evv251 PMC4758247 26660355

[tpj70919-bib-0004] Bierhorst, D.W. (1975) Gametophytes and embryos of *Actinostachys pennula*, *A. wagneri*, and *Schizaea elegans*, with notes on other species. American Journal of Botany, 62, 319–335.

[tpj70919-bib-0005] Bolger, A.M. , Lohse, M. & Usadel, B. (2014) Trimmomatic: a flexible trimmer for Illumina sequence data. Bioinformatics, 30, 2114–2120. 24695404 10.1093/bioinformatics/btu170 PMC4103590

[tpj70919-bib-0006] Borowiec, M.L. (2016) AMAS: a fast tool for alignment manipulation and computing of summary statistics. PeerJ, 4, e1660. 26835189 10.7717/peerj.1660 PMC4734057

[tpj70919-bib-0007] Boussardon, C. , Salone, V. , Avon, A. , Berthomé, R. , Hammani, K. , Okuda, K. et al. (2012) Two interacting proteins are necessary for the editing of the NdhD‐1 site in *Arabidopsis* plastids. Plant Cell, 24, 3684–3694. 23001034 10.1105/tpc.112.099507 PMC3480295

[tpj70919-bib-0008] Brown, J.W. , Walker, J.F. & Smith, S.A. (2017) Phyx: phylogenetic tools for unix. Bioinformatics, 33, 1886–1888. 28174903 10.1093/bioinformatics/btx063 PMC5870855

[tpj70919-bib-0009] Bushmanova, E. , Antipov, D. , Lapidus, A. & Prjibelski, A.D. (2019) rnaSPAdes: a *de novo* transcriptome assembler and its application to RNA‐seq data. GigaScience, 8, giz100. 31494669 10.1093/gigascience/giz100 PMC6736328

[tpj70919-bib-0076] Bushnell, B. (2014) BBMap: a fast, accurate, splice‐aware aligner. *9th Annual Genomics of Energy and Environment Meeting, Walnut Creek, CA, USA*.

[tpj70919-bib-0010] Chateigner‐Boutin, A.L. , Ramos Vega, M. , Guevara Garcia, A. , Andres, C. , de la Luz Gutierrez, M. , Nava, A. et al. (2008) CLB19, a pentatricopeptide repeat protein required for editing of *rpoA* and *clpP* chloroplast transcripts. Plant Journal, 56, 590–602. 10.1111/j.1365-313X.2008.03634.x 18657233

[tpj70919-bib-0011] Chateigner‐Boutin, A.L. & Small, I. (2011) Organellar RNA editing. Wiley Interdisciplinary Reviews: RNA, 2, 493–506. 21957039 10.1002/wrna.72

[tpj70919-bib-0012] Conway, J.R. , Lex, A. & Gehlenborg, N. (2017) UpSetR: an R package for the visualization of intersecting sets and their properties. Bioinformatics, 33, 2938–2940. 28645171 10.1093/bioinformatics/btx364 PMC5870712

[tpj70919-bib-0013] Covello, P.S. & Gray, M.W. (1993) On the evolution of RNA editing. Trends in Genetics, 9, 265–268. 8379005 10.1016/0168-9525(93)90011-6

[tpj70919-bib-0014] de Longevialle, A.F. , Meyer, E.H. , Andrés, C. , Taylor, N.L. , Lurin, C. , Millar, A.H. et al. (2007) The pentatricopeptide repeat gene OTP43 is required for trans‐splicing of the mitochondrial nad1 intron 1 in *Arabidopsis thaliana* . Plant Cell, 19, 3256–3265. 17965268 10.1105/tpc.107.054841 PMC2174710

[tpj70919-bib-0015] Dennis, M. , Low, S.Y. , Viljoen, A. , Pullakhandam, A. , Francs‐Small, C.C.d. , Campbell‐Clause, L. et al. (2025) GRASP: a modular toolkit for building synthetic pentatricopeptide repeat RNA‐binding proteins. *bioRxiv* . 10.1093/nar/gkaf1169 PMC12604669 41224122

[tpj70919-bib-0016] Dierckxsens, N. , Mardulyn, P. & Smits, G. (2017) NOVOPlasty: de novo assembly of organelle genomes from whole genome data. Nucleic Acids Research, 45, e18. 28204566 10.1093/nar/gkw955 PMC5389512

[tpj70919-bib-0017] Edera, A.A. & Sanchez‐Puerta, M.V. (2021) Computational detection of plant RNA editing events. Methods in Molecular Biology, 2181, 13–34. 32729072 10.1007/978-1-0716-0787-9_2

[tpj70919-bib-0018] Fauskee, B.D. , Kuo, L.Y. , Heath, T.A. , Xie, P.J. & Pryer, K.M. (2025) Comparative phylogenetic analyses of RNA editing in fern plastomes suggest possible adaptive innovations. New Phytologist, 247, 2945–2963. 40415590 10.1111/nph.70244

[tpj70919-bib-0019] Fauskee, B.D. , Sigel, E.M. , Pryer, K.M. & Grusz, A.L. (2021) Variation in frequency of plastid RNA editing within *Adiantum* (Pteridaceae) implies rapid evolution in fern plastomes. American Journal of Botany, 108, 2558–2571. 10.1002/ajb2.1649 33969475

[tpj70919-bib-0020] Funk, H.T. , Berg, S. , Krupinska, K. , Maier, U.G. & Krause, K. (2007) Complete DNA sequences of the plastid genomes of two parasitic flowering plant species, *Cuscuta reflexa* and *Cuscuta gronovii* . BMC Plant Biology, 7, 45. 17714582 10.1186/1471-2229-7-45 PMC2089061

[tpj70919-bib-0021] Gerke, P. , Szövényi, P. , Neubauer, A. , Lenz, H. , Gutmann, B. , McDowell, R. et al. (2020) Towards a plant model for enigmatic U‐to‐C RNA editing: the organelle genomes, transcriptomes, editomes and candidate RNA editing factors in the hornwort *Anthoceros agrestis* . New Phytologist, 225, 1974–1992. 31667843 10.1111/nph.16297

[tpj70919-bib-0022] Grewe, F. , Herres, S. , Viehöver, P. , Polsakiewicz, M. , Weisshaar, B. & Knoop, V. (2011) A unique transcriptome: 1782 positions of RNA editing alter 1406 codon identities in mitochondrial mRNAs of the lycophyte *Isoetes engelmannii* . Nucleic Acids Research, 39, 2890–2902. 21138958 10.1093/nar/gkq1227 PMC3074146

[tpj70919-bib-0023] Guo, W. , Grewe, F. & Mower, J.P. (2015) Variable frequency of plastid RNA editing among ferns and repeated loss of uridine‐to‐cytidine editing from vascular plants. PLoS One, 10, e0117075. 25568947 10.1371/journal.pone.0117075 PMC4287625

[tpj70919-bib-0024] Guo, W. , Zhu, A. , Fan, W. & Mower, J.P. (2017) Complete mitochondrial genomes from the ferns *Ophioglossum californicum* and *Psilotum nudum* are highly repetitive with the largest organellar introns. New Phytologist, 213, 391–403. 27539928 10.1111/nph.14135

[tpj70919-bib-0025] Gutmann, B. , Royan, S. , Schallenberg‐Rüdinger, M. , Lenz, H. , Castleden, I.R. , McDowell, R. et al. (2020) The expansion and diversification of pentatricopeptide repeat RNA‐editing factors in plants. Molecular Plant, 13, 215–230. 31760160 10.1016/j.molp.2019.11.002

[tpj70919-bib-0026] Hayes, M.L. , Garcia, E.T. , Chun, S.O. & Selke, M. (2024) Crosslinking of base‐modified RNAs by synthetic DYW‐KP base editors implicates an enzymatic lysine as the nitrogen donor for U‐to‐C editing. Journal of Biological Chemistry, 300, 107454. 38852885 10.1016/j.jbc.2024.107454 PMC11332814

[tpj70919-bib-0027] Huang, C.Y. , Grunheit, N. , Ahmadinejad, N. , Timmis, J.N. & Martin, W. (2005) Mutational decay and age of chloroplast and mitochondrial genomes transferred recently to angiosperm nuclear chromosomes. Plant Physiology, 138, 1723–1733. 15951485 10.1104/pp.105.060327 PMC1176441

[tpj70919-bib-0028] Huynh, S.D. , Melonek, J. , Colas des Francs‐Small, C. , Bond, C.S. & Small, I. (2023) A unique C‐terminal domain contributes to the molecular function of restorer‐of‐fertility proteins in plant mitochondria. New Phytologist, 240, 830–845. 37551058 10.1111/nph.19166

[tpj70919-bib-0029] Ichinose, M. & Sugita, M. (2017) RNA editing and its molecular mechanism in plant organelles. Genes, 8, 5. 10.3390/genes8010005 PMC5295000 28025543

[tpj70919-bib-0030] Ishibashi, K. , Small, I. & Shikanai, T. (2019) Evolutionary model of plastidial RNA editing in angiosperms presumed from genome‐wide analysis of *Amborella trichopoda* . Plant and Cell Physiology, 60, 2141–2151. 31150097 10.1093/pcp/pcz111

[tpj70919-bib-0031] Jansen, R.K. & Ruhlman, T.A. (2012) Plastid genomes of seed plants. In: Genomics of chloroplasts and mitochondria. Dordrecht, Netherlands: Springer, pp. 103–126.

[tpj70919-bib-0032] Jombart, T. , Balloux, F. & Dray, S. (2010) Adephylo: new tools for investigating the phylogenetic signal in biological traits. Bioinformatics, 26, 1907–1909. 20525823 10.1093/bioinformatics/btq292

[tpj70919-bib-0033] Katoh, K. & Standley, D.M. (2013) MAFFT multiple sequence alignment software version 7: improvements in performance and usability. Molecular Biology and Evolution, 30, 772–780. 23329690 10.1093/molbev/mst010 PMC3603318

[tpj70919-bib-0034] Ke, B.F. , Wang, G.J. , Labiak, P.H. , Rouhan, G. , Chen, C.W. , Shepherd, L.D. et al. (2022) Systematics and plastome evolution in Schizaeaceae. Frontiers in Plant Science, 13, 885501. 35909781 10.3389/fpls.2022.885501 PMC9328107

[tpj70919-bib-0035] Kearse, M. , Moir, R. , Wilson, A. , Stones‐Havas, S. , Cheung, M. , Sturrock, S. et al. (2012) Geneious basic: an integrated and extendable desktop software platform for the organization and analysis of sequence data. Bioinformatics, 28, 1647–1649. 22543367 10.1093/bioinformatics/bts199 PMC3371832

[tpj70919-bib-0036] Khanna, A. , Larson, D.E. , Srivatsan, S.N. , Mosoir, M. , Abbott, T.E. , Kiwala, S. et al. (2022) Bam‐readcount—rapid generation of basepair‐resolution sequence metrics. The Journal of Open Source Software, 7, 3722. 10.21105/joss.03722 PMC13367885 42453900

[tpj70919-bib-0037] Knie, N. , Grewe, F. , Fischer, S. & Knoop, V. (2016) Reverse U‐to‐C RNA editing exceeds C‐to‐U RNA editing in some ferns—a monilophyte‐wide comparison of chloroplast and mitochondrial RNA editing suggests independent evolution of the two processes in both organelles. BMC Evolutionary Biology, 16, 134. 27329857 10.1186/s12862-016-0707-z PMC4915041

[tpj70919-bib-0038] Knoop, V. (2023) RNA editing in plant organelles and beyond. Journal of Experimental Botany, 74, 2273–2294. 36527364 10.1093/jxb/erac488

[tpj70919-bib-0039] Kosakovsky Pond, S.L. , Poon, A.F. , Velazquez, R. , Weaver, S. , Hepler, N.L. , Murrell, B. et al. (2020) HyPhy 2.5—a customizable platform for evolutionary hypothesis testing using phylogenies. Molecular Biology and Evolution, 37, 295–299. 31504749 10.1093/molbev/msz197 PMC8204705

[tpj70919-bib-0040] Krieg, C.P. & Chambers, S.M. (2022) The ecology and physiology of fern gametophytes: a methodological synthesis. Applications in Plant Sciences, 10, e11464. 35495196 10.1002/aps3.11464 PMC9039797

[tpj70919-bib-0041] Kugita, M. , Yamamoto, Y. , Fujikawa, T. , Matsumoto, T. & Yoshinaga, K. (2003) RNA editing in hornwort chloroplasts makes more than half the genes functional. Nucleic Acids Research, 31, 2417–2423. 12711687 10.1093/nar/gkg327 PMC154213

[tpj70919-bib-0042] Kwok van der Giezen, F.M. , McDowell, R. , Duncan, O. , Zumkeller, S. , Colas des Francs‐Small, C. & Small, I. (2025) High conservation of translation‐enabling RNA editing sites in hyper‐editing ferns implies they are not selectively neutral. Molecular Biology and Evolution, 42, msaf241. 41024750 10.1093/molbev/msaf241 PMC12548569

[tpj70919-bib-0043] Kwok van der Giezen, F.M. , Viljoen, A. , Campbell‐Clause, L. , Dao, N.T. , Colas des Francs‐Small, C. & Small, I. (2024) Insights into U‐to‐C RNA editing from the lycophyte *Phylloglossum drummondii* . The Plant Journal, 119, 445–459. 38652016 10.1111/tpj.16775

[tpj70919-bib-0044] Labiak, P.H. & Karol, K.G. (2017) Plastome sequences of an ancient fern lineage reveal remarkable changes in gene content and architecture. American Journal of Botany, 104, 1008–1018. 28754764 10.3732/ajb.1700135

[tpj70919-bib-0045] Langmead, B. & Salzburg, S.L. (2012) Fast gapped‐read alignment with bowtie 2. Nature Methods, 9, 357–359. 22388286 10.1038/nmeth.1923 PMC3322381

[tpj70919-bib-0046] Lesch, E.M. , Schilling, T. , Brenner, S. , Yang, Y. , Gruss, O.J. , Knoop, V. et al. (2022) Plant mitochondrial RNA editing factors can perform targeted C‐to‐U editing of nuclear transcripts in human cells. Nucleic Acids Research, 50, 9966–9983. 36107771 10.1093/nar/gkac752 PMC9508816

[tpj70919-bib-0047] Li, F.‐W. , Brouwer, P. , Carretero‐Paulet, L. , Cheng, S. , de Vries, J. , Delaux, P.‐M. et al. (2018) Fern genomes elucidate land plant evolution and cyanobacterial symbioses. Nature Plants, 4, 460–472. 29967517 10.1038/s41477-018-0188-8 PMC6786969

[tpj70919-bib-0048] Li, F.‐W. , Kuo, L.‐Y. , Pryer, K.M. & Rothfels, C.J. (2016) Genes translocated into the plastid inverted repeat show decelerated substitution rates and elevated GC content. Genome Biology and Evolution, 8, 2452–2458. 27401175 10.1093/gbe/evw167 PMC5010901

[tpj70919-bib-0049] Li, H. , Handsaker, B. , Wysoker, A. , Fennell, T. , Ruan, J. , Homer, N. et al. (2009) The sequence alignment/map format and SAMtools. Bioinformatics, 25, 2078–2079. 19505943 10.1093/bioinformatics/btp352 PMC2723002

[tpj70919-bib-0050] Li, L. , Gu, X. , Lu, C. , Liang, Y. , Ping, J. , Su, Y. et al. (2025) Identification of MORF family in ferns. Molecular regulation of organellar RNA editing in *Osmunda japonica* and *Plenasium vachellii* . Biology, 14, 1463. 41154865 10.3390/biology14101463 PMC12561531

[tpj70919-bib-0051] Lukeš, J. , Archibald, J.M. , Keeling, P.J. , Doolittle, W.F. & Gray, M.W. (2011) How a neutral ratchet can build cellular complexity. IUBMB Life, 63, 528–537. 21698757 10.1002/iub.489

[tpj70919-bib-0052] Lurin, C. , Andrés, C. , Aubourg, S. , Bellaoui, M. , Bitton, F. , Bruyère, C. et al. (2004) Genome‐wide analysis of *Arabidopsis* pentatricopeptide repeat proteins reveals their essential role in organelle biogenesis. Plant Cell, 16, 2089–2103. 15269332 10.1105/tpc.104.022236 PMC519200

[tpj70919-bib-0053] Minh, B.Q. , Schmidt, H.A. , Chernomor, O. , Schrempf, D. , Woodhams, M.D. , von Haeseler, A. et al. (2020) IQ‐TREE 2: new models and efficient methods for phylogenetic inference in the genomic era. Molecular Biology and Evolution, 37, 1530–1534. 32011700 10.1093/molbev/msaa015 PMC7182206

[tpj70919-bib-0054] Mower, J.P. (2008) Modeling sites of RNA editing as a fifth nucleotide state reveals progressive loss of edited sites from angiosperm mitochondria. Molecular Biology and Evolution, 25, 52–61. 17940211 10.1093/molbev/msm226

[tpj70919-bib-0055] Mower, J.P. & Vickrey, T.L. (2018) Structural diversity among plastid genomes of land plants. Advances in Botanical Research, 85, 263–292.

[tpj70919-bib-0056] Oldenkott, B. , Tang, Y. , Lesch, E. , Knoop, V. & Shallenberg‐Rüdinger, M. (2019) Plant‐type pentatricopeptide repeat proteins with a DYW domain drive C‐to‐U RNA editing in *Escherichia coli* . Communications Biology, 2, 85. 30854477 10.1038/s42003-019-0328-3 PMC6397227

[tpj70919-bib-0075] Pelosi, J.A. , Davenport, R. , Barbazuk, W.B. , Sessa, E.B. & Kuo, L.‐Y. (2024) An efficient and effective RNA extraction protocol for ferns. Applications in Plant Sciences, 12, e11617. 39628544 10.1002/aps3.11617 PMC11610414

[tpj70919-bib-0057] PPG I . (2016) A community‐derived classification for extant lycophytes and ferns. Journal of Systematics and Evolution, 54, 563–603.

[tpj70919-bib-0058] Prikryl, J. , Rojas, M. , Schuster, G. & Barkan, A. (2011) Mechanism of RNA stabilization and translational activation by a pentatricopeptide repeat protein. Proceedings of the National Academy of Sciences, 108, 415–420. 10.1073/pnas.1012076108 PMC3017144 21173259

[tpj70919-bib-0077] R Core Team . (2021) R: a language and environment for statistical computing. Vienna, Austria: R Foundation for Statistical Computing. Available from: https://www.R-project.org/

[tpj70919-bib-0059] Rochaix, J.D. (1978) Restriction endonuclease map of the chloroplast DNA of *Chlamydomonas reinhardii* . Journal of Molecular Biology, 126, 597–617. 745241 10.1016/0022-2836(78)90011-6

[tpj70919-bib-0060] Royan, S. , Gutmann, B. , Colas des Francs‐Small, C. , Honkanen, S. , Schmidberger, J. , Soet, A. et al. (2021) A synthetic RNA editing factor edits its target site in chloroplasts and bacteria. Communications Biology, 4, 545. 33972654 10.1038/s42003-021-02062-9 PMC8110955

[tpj70919-bib-0061] Rüdinger, M. , Polsakiewicz, M. & Knoop, V. (2008) Organellar RNA editing and plant‐specific extensions of pentatricopeptide repeat proteins in jungermanniid but not in marchantiid liverworts. Molecular Biology and Evolution, 25, 1405–1414. 18400790 10.1093/molbev/msn084

[tpj70919-bib-0062] Ruhlman, T.A. & Jansen, R.K. (2014) The plastid genomes of flowering plants. In: Chloroplast biotechnology: methods and protocols. Totowa, New Jersey, USA: Humana Press, pp. 3–38. 10.1007/978-1-62703-995-6_1 24599844

[tpj70919-bib-0063] Shen, C. , Xu, H. , Huang, W.H. , Zhao, Q. & Zhu, R.L. (2024) Is RNA editing truly absent in the complex thalloid liverworts (Marchantiopsida)? Evidence of extensive editing from *Cyathodium cavernarum* . New Phytologist, 242, 2817–2831. 38587065 10.1111/nph.19750

[tpj70919-bib-0064] Simão, F.A. , Waterhouse, R.M. , Ioannidis, P. , Kriventseva, E.V. & Zdobnov, E.M. (2015) BUSCO: assessing genome assembly and annotation completeness with single‐copy orthologs. Bioinformatics, 31, 3210–3212. 26059717 10.1093/bioinformatics/btv351

[tpj70919-bib-0065] Small, I.D. , Schallenberg‐Rüdinger, M. , Takenaka, M. , Mireau, H. & Ostersetzer‐Biran, O. (2020) Plant organellar RNA editing: what 30 years of research has revealed. The Plant Journal, 101, 1040–1056. 31630458 10.1111/tpj.14578

[tpj70919-bib-0066] Tillich, M. , Lehwark, P. , Morton, B.R. & Maier, U.G. (2006) The evolution of chloroplast RNA editing. Molecular Biology and Evolution, 23, 1912–1921. 16835291 10.1093/molbev/msl054

[tpj70919-bib-0067] Villarreal, J.C. , Turmel, M. , Bourgouin‐Couture, M. , Laroche, J. , Salazar Allen, N. , Li, F.‐W. et al. (2018) Genome‐wide organellar analyses from the hornwort *Leiosporoceros dussii* show low frequency of RNA editing. PLoS One, 13, e0200491. 30089117 10.1371/journal.pone.0200491 PMC6082510

[tpj70919-bib-0068] Walker, B.J. , Abeel, T. , Shea, T. , Priest, M. , Abouelliel, A. , Sakthikumar, S. et al. (2014) Pilon: an integrated tool for comprehensive microbial variant detection and genome assembly improvement. PLoS One, 9, e112963. 25409509 10.1371/journal.pone.0112963 PMC4237348

[tpj70919-bib-0069] Wertheim, J.O. , Murrell, B. , Smith, M.D. , Kosakovsky Pond, S.L. & Scheffler, K. (2015) RELAX: detecting relaxed selection in a phylogenetic framework. Molecular Biology and Evolution, 32, 820–832. 25540451 10.1093/molbev/msu400 PMC4327161

[tpj70919-bib-0070] Wicke, S. , Müller, K.F. , DePamphilis, C.W. , Quandt, D. , Bellot, S. & Schneeweiss, G.M. (2016) Mechanistic model of evolutionary rate variation en route to a nonphotosynthetic lifestyle in plants. Proceedings of the National Academy of Sciences, 113, 9045–9050. 10.1073/pnas.1607576113 PMC4987836 27450087

[tpj70919-bib-0071] Wicke, S. , Schneeweiss, G.M. , dePamphilis, C.W. , Müller, K.F. & Quandt, D. (2011) The evolution of the plastid chromosome in land plants: gene content, gene order, gene function. Plant Molecular Biology, 76, 273–297. 21424877 10.1007/s11103-011-9762-4 PMC3104136

[tpj70919-bib-0072] Wolf, P.G. , Rowe, C.A. & Hasebe, M. (2004) High levels of RNA editing in a vascular plant chloroplast genome: analysis of transcripts from the fern *Adiantum capillus‐veneris* . Gene, 339, 89–97. 15363849 10.1016/j.gene.2004.06.018

[tpj70919-bib-0073] Zhu, A. , Guo, W. , Gupta, S. , Fan, W. & Mower, J.P. (2016) Evolutionary dynamics of the plastid inverted repeat: the effects of expansion, contraction, and loss on substitution rates. New Phytologist, 209, 1747–1756. 26574731 10.1111/nph.13743

[tpj70919-bib-0074] Zumkeller, S. , Polsakiewicz, M. & Knoop, V. (2023) Rickettsial DNA and a trans‐splicing rRNA group I intron in the unorthodox mitogenome of the fern *Haplopteris ensiformis* . Communications Biology, 6, 296. 36941328 10.1038/s42003-023-04659-8 PMC10027690

