# Supplementary material for: Losing genes, gaining edits: how relaxed selection and inverted repeat expansion shape RNA editing in Schizaeaceae plastomes
Source: Plant J. 2026 Jun 12;126(5):e70919. doi: 10.1111/tpj.70919 (PMC13263110; doi:10.1111/tpj.70919)
Supplement: Supplementary file 1 — Appendix S1. Voucher information and data accession numbers for species studied here. Accession numbers for raw genomic and transcriptomic reads are available along with accession numbers for chloroplast genome assemblies. [file TPJ-126-0-s001.docx]

| **Species** | **Voucher Collection No.** | **Locality** | **Plastome NCBI Accession No.** | **DNA reads SRA Accession** | **RNA reads SRA Accession** |
| --- | --- | --- | --- | --- | --- |
| *Lygodium microphyllum* | Lu30898 | Mt. Tanfeng, Tapiei City, Taiwan | PX693348 | SRR36332479 | SRR36317039 |
| *Anemia phyllitidis* | Kuo 4249 | Cecilia Koo Botanic Conservation Center, Taiwan | PV938982 | SRR34530197 | SRR34528657 |
| *Actinostachys digitata* | Kuo 4574-3 | Syuhai Grassland, Mudan Township. Pingtung County, Taiwan | PX693347 | SRR36330971 | SRR36330087 |
| *Schizaea dichotoma* | Kuo 4570 | Tien Pond, Lanyu Township, Taitung County, Taiwan | PX693349 | SRR36332478 | SRR36332038 |
